# Evaluation of the use of GRADE in dentistry systematic reviews and its impact on conclusions: a protocol for a methodological study

**DOI:** 10.1186/s13643-023-02199-0

**Published:** 2023-03-13

**Authors:** Sara Ibrahim, Maria Azab, Anna Miroshnychenko, Romina Brignardello-Petersen

**Affiliations:** 1 Department of Health Research Methods, Evidence, and Impact, McMaster University, 1280 Main St West, Hamilton, Ontario L8S 4L8 Canada; 2 Temerty Faculty of Medicine, University of Toronto, 3359 Mississauga Rd, Mississauga, Ontario L5L 1C6 Canada

**Keywords:** Dentistry, Systematic review, GRADE, Conclusions, Research methodology

## Abstract

**Background:**

There is a growing body of evidence of systematic reviews (SRs) with varying degrees of methodological quality. The Grading of Recommendations, Assessment, Development, and Evaluation (GRADE) approach allows SR authors to assess the certainty of the evidence they found and transparently relay their conclusions. As there appears to be infrequent utilization of GRADE in the field of dentistry, to identify the impact of GRADE, the aim of this study is to evaluate the use of GRADE in the dental literature and determine whether SRs that use GRADE differ from those that do not with respect to their conclusions.

**Methods/design:**

We will search Ovid MEDLINE for SRs published from 2016 to the present. We will conduct both screening and data extraction independently and in duplicate and use pre-piloted, standardized forms for data extraction. We will determine the frequency of the use of GRADE and the varying levels of certainty in the current literature and evaluate whether GRADE is being used appropriately. We will also evaluate whether SRs not using GRADE differ from those that use GRADE with regard to methodological quality. We will also determine whether the conclusions of SRs that do not use GRADE would change had GRADE been utilized. Additionally, we will evaluate whether SRs using GRADE are more likely to formulate appropriate conclusions compared to SRs that do not use it.

**Discussion:**

This study will investigate the frequency of GRADE assessments in dentistry SRs and the impact of GRADE assessments on the conclusions of a SR. It has important implications for both SR authors and users of this type of literature.

**Supplementary Information:**

The online version contains supplementary material available at 10.1186/s13643-023-02199-0.

## Background

Systematic reviews (SRs) and meta-analyses synthesize information from a variety of studies and are hence often considered at the top of the hierarchy of evidence-based medical research [1]. Over the years, there has been a rapid increase in SR publications [2, 3]. For example, a PubMed search of SRs results in 5261 potential reviews published in 2010 and 27,915 in 2020. One study evaluated the current biomedical SRs published and indexed in PubMed and estimated that only a small minority appear to be methodologically sound and informative while the vast majority appears to be either flawed or redundant and not useful [3].

A recent study evaluating the methodological quality and risk of bias (RoB) in SRs relevant to orthodontics found that only 56% of the SRs in their sample were judged as having low RoB when assessed with the Risk of Bias Assessment Tool for Systematic Reviews (ROBIS) [4]. They also found that less than 50% of the SRs were rated at a good methodological quality level when using the Assessing the Methodological Quality of Systematic Reviews (AMSTAR) tool [4]. Similarly, a study investigating the methodological quality of SRs of treatments for peri-implantitis found that out of the 23 included SRs, 6 had low and 14 had critically low methodological quality, as judged by an AMSTAR assessment, and all but one had a high risk of bias according to the ROBIS tool [5].

One of the characteristics of a high-quality, trustworthy SR is an assessment of the overall quality of the evidence [1]. The Grading of Recommendations, Assessment, Development, and Evaluation (GRADE) approach is one tool to conduct such assessments [6]. GRADE guidelines were first published in 2008 and followed by an additional series of publications for SR authors in 2011 [7]. This approach allows for systematic reviewers to conduct assessments of certainty of evidence (also known as quality of evidence) and then present these results in a transparent and structured manner [6]. It is used to rate the certainty of the evidence, per outcome, across all studies included in a SR [6]. To begin, SR authors must determine the initial certainty of the evidence, SRs of randomized controlled trials (RCTs) start at high certainty while SRs of observational studies starts at low certainty. Five factors can decrease the certainty of the evidence (study limitations (RoB), imprecision, inconsistency, indirectness, publication bias), and in the case of SRs of observational studies, three factors can increase the certainty of the evidence (large magnitude of effect, dose-response effect, confounders would likely minimize the effect or increase it if the results found no effect) [6]. By considering these domains, SR authors can reach a final rating of either high, moderate, low, or very low certainty of evidence [6].

Certainty of evidence assessments allow for a more transparent and holistic presentation of the conclusions of SRs that focus more on clinical importance rather than statistical significance [6]. The GRADE approach has become widely accepted among professional bodies, medical journals, and healthcare regulatory authorities [8]. However, the use of GRADE in dentistry has been seemingly less frequent, with one study reporting that only 27 of 91 oral health SRs published between 2008 and 2013 utilized GRADE [8, 9]. Furthermore, a methodological study revealed that only 61.4% of oral health SRs assessed the risk of bias in the included studies [10]. Since risk of bias is a key domain of the GRADE approach, this suggests that the use of GRADE is relatively infrequent in the dental literature [9, 10].

This is a protocol for a methodological study which aims to determine the frequency of SRs in the field of dentistry which conducts GRADE assessments and to determine whether the use of GRADE changes the conclusions of dentistry SRs which do not utilize the tool.

## Methods and analysis

We will conduct two studies to assess the frequency (objective 1) and the implications (objective 2) of the use of GRADE in the current dental literature. We adhered to all sections of the Preferred Reporting Items for Systematic Reviews and Meta-Analyses Protocols (PRISMA-P) statement that applied to our methodological study (see Additional file 1) [11].

### Search strategy

We will utilize one search strategy to retrieve potentially eligible SRs for both studies.

We will perform a search in Ovid MEDLINE from January 1, 2016, to the present day. We will use search filters from the Health Information Research Unit (HIRU) of McMaster University as well as the Medical Subject Heading (MeSH) “dentistry” to search for SRs [12]. There will be no language restrictions in our search strategy. Our final search strategy (Table 1) will be reviewed by a methods expert (R.B.-P.).

**Table 1:** Ovid MEDLINE search

1. MEDLINE.tw.	
2. systematic review.tw.	
3. meta analysis.pt.	
4. 1 or 2 or 3^a^	
5. exp Dentistry/	
6. 4 and 5	
7. limit 6 to yr= “2016 -Current”	

^a^Terms 1–4 refer to the HIRU review filter that maximizes specificity

### Screening process

For both studies, we will screen the titles/abstracts and full texts of the retrieved citations independently and in duplicate using Covidence. Conflicts will be resolved through discussion or by a third reviewer when necessary. Eligibility criteria will be different for each study and are described below.

### Study sample and random sampling of citations

We will screen all citations retrieved from our search in MEDLINE at the title and abstract screening stage. We will then assign a random number to each title and abstract meeting the inclusion criteria using Microsoft Excel. We will complete full-text screening according to the random number assigned to each study starting from the lowest number and working in ascending order until the number of included full-text meets our target sample size.

To obtain an informative sample, we aim to include a minimum of 50 SRs that use GRADE. Given the findings of a previous study which found that nearly 30% of oral health SRs used GRADE, we used a more conservative estimate of 25% and determined that our target sample size should be 200 SRs to allow for at least 50 SRs using GRADE [9].

### Study 1: Assessment of the frequency of the utilization of the GRADE approach in the recent dental literature

The following are the objectives:

A. To determine the frequency of the utilization of GRADE in dentistry SRs
B. To summarize the frequency of the levels of certainty determined by GRADE assessments conducted in dentistry SRs
C. To assess whether GRADE is being used appropriately at both the review and outcome level (for the primary outcome) in dentistry SRs

To evaluate whether SRs using GRADE differ from those that do not use GRADE with regard to methodological quality

#### Eligibility criteria

##### Inclusion criteria

We will include SRs of interventions in dentistry, published in English, which included only RCTs.

We will consider SRs to be studies in which either one of the two following criteria are met:

1. The authors refer to the study as either a SR or meta-analysis and search at least one electronic database for published studies.
2. The authors search at least one electronic database for published studies and use well-defined eligibility criteria. We will consider eligibility criteria to be well-defined if it comments on all of the following:
  - The study designs to be included in the SR
  - The population of interest for the research question (e.g., patient characteristics, specific indication for treatment)
  - The intervention(s)/comparator(s) the authors aim to investigate

In order to be considered a SR in dentistry, one of the following conditions must be met:

- The SR includes studies in which patients receive treatment for an oral pathology or undergo an oral health-related procedure.
- The SR includes studies in which one oral health-related intervention is compared to another, placebo, or standard care.

##### Exclusion criteria

- SRs which conduct network meta-analyses (NMAs)
- SRs which find no evidence and therefore fail to include any studies
- SRs which are published in combination with another type of study (e.g., case study/series, health technology assessment, clinical practice guidelines)

#### Data extraction

Pairs of reviewers will extract data from eligible studies independently and in duplicate using forms created in Microsoft Excel. Reviewers will undergo a data extraction calibration exercise of three SRs per reviewer and pilot the standardized extraction sheet prior to the start of extraction. We will resolve conflicts through discussion or by consulting a third reviewer.

Data to be extracted from each SR will include general characteristics including title, author(s), journal, year of publication, country of authors, and, if applicable, dentistry specialty or specialties [13]. For SRs conducting GRADE assessments, reviewers will also identify the primary outcome of each SR, which is the outcome defined as such by the authors or the outcome first listed in the methods section. If there are multiple primary outcomes defined by the SR authors, we will use the first outcome mentioned. If the methods section does not clearly describe the outcomes, we will consider the first outcome mentioned in the results section to be the primary outcome. Additionally, if a review assesses multiple comparisons for the primary outcome, we will only consider the results of the first comparison described in the results. If the primary outcome is assessed at multiple time points, we will consider only the results of the shortest time point. We will also extract data on the methodology of each SR, including the methods of searching, screening, and data extraction as well as the results of the SRs, including the outcomes analyzed and the number of included RCTs. In order to allow us to select an outcome of interest for study 2, we will also extract data on whether the SR authors conducted and reported the results of an RoB assessment and whether they report the number of participants analyzed for narratively reported outcomes.

Regarding GRADE, we will extract the extent to which it was used in each SR (for all, some, or none of its outcomes), whether summary of findings tables were used, whether GRADE was used for all outcomes that were meta-analyzed, and whether GRADE was used for outcomes that were not meta-analyzed. We will also determine whether the SR authors refrain from making recommendations or statements about whether an intervention should or should not be used in clinical practice. We will search for potential recommendations in the conclusion, discussion, and abstract of the SR. We will extract additional data on the GRADE assessments for the primary outcome including the final certainty of the evidence rating, ratings and explanations for each GRADE domain, and additional information to allow us to determine whether GRADE was used appropriately at the outcome level. We will note any other issues with the GRADE assessments of the SR authors as part of our evaluation of whether GRADE was used appropriately.

We will also extract whether GRADE assessments were incorporated into conclusions about the primary outcome in the abstract and body of the SR. We will define a conclusion as a statement in which the authors interpret their results by stating whether the intervention(s) has beneficial or harmful effects relative to, or is no different from, the comparator(s) or stating that there is a lack of evidence regarding the outcome. We will first extract the conclusions about the primary outcome from the abstract. If there is no conclusion section in the abstract, we will extract any conclusion statements from the results of the abstract. We will also extract conclusions about the primary outcome from the body of the SR, referring to the SRs designated conclusion section to minimize subjective judgments. If there is no conclusion section, we will extract the conclusion from the discussion section. Finally, if there is no clear conclusion statement in any of the aforementioned sections, we will not assess the conclusions of the SR but will still incorporate the SR in our other analyses (e.g., percentage of use of GRADE).

A summary of the data extraction fields can be found in Table 2. Should further data necessitate extraction, we will modify the standardized form, extract this new data for all eligible studies, and report these protocol modifications in the final publication.

**Table 2:** Data extraction fields

Section	Data to be extracted
Abstract	• Verbatim quotation of the conclusion statements from the abstract pertaining to the primary outcome ◦ If there is no conclusion section in the abstract, we will extract this from the results of the abstract. • For studies using GRADE, are the GRADE certainty of the evidence ratings incorporated in the conclusion statements pertaining to the primary outcome in the abstract? (yes/no)
SR characteristics	• Title of the SR • Last name of the first author • Corresponding author’s name and email • Journal of publication • Year of publication • Country of authors
Methods	• Specialty of dentistry (indicate all that apply according to the definitions of the American Dental Association) [13] ◦ If the SR investigates general dentistry interventions (e.g., tooth brushing), this is not applicable • Primary outcome (for studies using GRADE) • Did the SR authors search for grey literature? (yes/no) • Was any aspect of screening conducted independently and in duplicate or by a single reviewer whose work was checked by another reviewer? (yes/no) • Was data extraction conducted independently and in duplicate or by a single reviewer whose work was checked by another reviewer? (yes/no)
Results	• Number of RCTs included overall • List of outcomes analyzed • Did the SR authors conduct meta-analyses? (yes/no/for some outcomes only) ◦ Specify any outcomes not meta-analyzed and provide the number of participants analyzed for that outcome ▪ Was GRADE used for any of these outcomes? • Does the SR use GRADE? (yes/no/for some outcomes only) • Does the SR assess RoB and report the findings of the RoB assessment? (yes/no) ◦ To answer yes to this question, the SR authors must, at minimum, indicate the overall risk of bias rating for each of the included RCTs. • If GRADE was used: ◦ Was GRADE used for all outcomes for which a meta-analysis was conducted? (yes/no) ◦ Are the GRADE assessments compiled in a summary of findings table? (yes/no) ◦ Do the SR authors refrain from making recommendations? (yes/no) ▪ If the SR authors make recommendations, copy and paste the verbatim quote of these recommendation statements. ◦ If GRADE was used for the primary outcome: ▪ Final certainty of the evidence for the primary outcome ▪ Were all five GRADE domains assessed for the primary outcome? (yes/no) ▪ List the ratings and explanations for the individual GRADE domains ▪ If the certainty of the evidence was downgraded: • Was an explanation provided for all domains that were downgraded? (yes/no) • Were the explanations informative? (yes/no) • For the GRADE domains which were downgraded, is there evidence that the SR authors assessed the domains using the incorrect criteria? (yes/no) ◦ If yes, copy and paste the corresponding explanation or provide a rationale for your judgment. ▪ Did the SR authors refrain from using the criteria for rating up when assessing the primary outcome? (yes/no) ▪ If the primary outcome is dichotomous, do the SR authors transform relative estimates of effect to absolute estimates of effect in order to assess imprecision? (yes/no) ◦ List any other issues with the SRs GRADE assessments.
Conclusions	• Verbatim quotation of the SR conclusion statements pertaining to the primary outcome from the SRs conclusion section ◦ If there is no conclusion section, this is to be extracted from the discussion section. If there is no conclusion outlined in the discussion, we will make a note that no conclusion was reported in the body of the SR. • For studies using GRADE, are the GRADE certainty of the evidence ratings incorporated in the conclusion statements pertaining to the primary outcome? (yes/no)

#### Data analysis

All retrieved articles will be presented in a study selection flow chart and the data of eligible studies summarized in tables.

*For determining how frequently GRADE is used in dentistry SRs (objective A)*, we will first conduct a descriptive analysis. We will calculate the percentage of SRs using GRADE overall, by year, and by dental specialty from our entire sample of studies. Additionally, for SRs using GRADE for at least one outcome, we will calculate the percentage of SRs using GRADE for outcomes in which no meta-analysis was conducted [14]. Finally, we will determine how frequently the authors incorporate GRADE into the conclusions of the SRs’ primary outcome in both the body of the SR and the abstract.

*For summarizing the frequency of the levels of certainty determined by GRADE assessments in dentistry SRs (objective B)*, we will determine the percentage of high, moderate, low, and very low certainty evidence among the primary outcomes of each SR and stratified by dental specialty. To evaluate which limitations are more likely to lead to lower certainty evidence in the current literature, we will also quantify the frequency of concerns that lead to rating down the certainty of the evidence for each GRADE domain.

*For assessing whether GRADE is being used appropriately (objective C)*, we will conduct two separate evaluations: at the review level and at the outcome level for the primary outcome of each SR [6, 15–20]. We will determine the percentage of SRs using GRADE appropriately at each level using the criteria outlined in Table 3 [6, 15–20].

**Table 3:** Checklist for determining whether GRADE was used appropriately

If the response to all of the following questions is “yes,” then GRADE has been used appropriately at the review level. If any of these criteria are not met, then GRADE was not used appropriately.	• Do the SR authors use GRADE for all outcomes for which a meta-analysis was conducted?
	• Are the GRADE assessments compiled in a GRADE evidence table (summary of findings table or evidence profile)?^a^
	• Do the SR authors refrain from making recommendations?
If the response to all of the following questions is “yes,” then GRADE has been used appropriately at the outcome-level. If any of these criteria are not met, then GRADE was not used appropriately.	• Are all five GRADE domains assessed?
	• Do the SR authors refrain from using the criteria for rating up?
	• Are explanations provided for all domains that are downgraded?
	• Are all the explanations for the downgraded domains informative?^b^ [15] ◦ For study limitations, do the authors indicate the proportion of studies that were at a concern for high risk of bias or the specific RoB assessment criteria that was of most concern? ◦ For imprecision, do the authors indicate whether the sample size or number of events was too low or whether the bounds of the CI have different meanings based on thresholds for the optimal information size or the effect size, respectively? ◦ For inconsistency, do the authors indicate how heterogeneity was judged (e.g., confidence interval overlap, statistical tests)? ◦ For indirectness, do the authors indicate whether it was the population, intervention, comparator, or outcome of the included RCTs that does not align with the SR question and is therefore a reason for concern? ◦ For publication bias, do the authors indicate the reason to suspect publication bias (e.g., funnel plot, suspected selective reporting)?
	• If the primary outcome is dichotomous, do the SR authors transform relative estimates of effects to absolute estimates in order to assess imprecision?
	• For the GRADE domains which were downgraded, is there evidence that the SR authors assessed the domains using the incorrect criteria (e.g, referring to the criteria for indirectness in the explanation for rating down imprecision, creating concerns for whether imprecision and indirectness were appropriately assessed)?

We will also note any other issues with the SRs GRADE assessments. If the review has any issues in the GRADE assessments at the review level or the outcome level, we will conclude that GRADE was not used appropriately at the review level or outcome level, respectively.

^a^We will consider any table that lists the certainty of the evidence ratings achieved after the GRADE assessments with information regarding which domains were downgraded (either reported in the table or in the footnotes) to meet this criterion.

^b^We will capture whether only some of the downgraded domains have informative explanations and report which domains were most or least likely to have informative explanations as defined above.

*We will evaluate whether SRs using GRADE differ from those that do not use GRADE with regard to methodological quality (objective D)*. To evaluate the methodological quality of each SR, we will refer to two aspects of the ROBIS tool [21]. First, we will determine whether the search strategy was comprehensive. A search strategy will be considered comprehensive if it searches for published and unpublished reports (by specifying grey literature databases or searching for unpublished reports through any other means) (ROBIS question 2.1) [21]. Second, we will assess whether efforts were made to minimize errors during screening (i.e., title/abstract and/or full-text screening) as well as data extraction (ROBIS questions 2.5, 3.1) [21]. As we anticipate poor reporting of the methods used for screening, we will consider any mention of conducting screening independently and in duplicate or by having a second reviewer check the work of another to be minimizing errors. For data extraction, as described in the ROBIS tool, SRs for which this process is conducted independently and in duplicate or by having a second reviewer check the work of another reviewer in detail will be considered to be minimizing errors [21]. We have chosen to utilize these two domains of ROBIS to maximize objectivity as the remaining domains require more judgment contextualized to each SRs clinical question. We will use the odds ratio and its 95% confidence interval (CI) to determine whether SRs using GRADE are more likely to (1) have a comprehensive search strategy that considers grey literature and (2) take steps to avoid errors in screening and data extraction.

### Study 2: Impact of the presence of GRADE assessments on the conclusions of dentistry-related systematic reviews

The following are the objectives:

A. To determine whether a lack of certainty of the evidence assessments is a predictor of inappropriately formulated conclusions in SRs
B. To determine whether the use of GRADE changes the conclusions of dentistry SRs which do not utilize the tool

#### Outcome of interest

To conduct this study, we will focus on a specific outcome across all SRs. We will determine the outcome of interest based on the following criteria:

1. The outcome of interest will be the outcome most frequently reported within our sample of SRs and for which the following information is also available:
  - The findings of the RoB assessment conducted by SR authors
  - The effect estimate with its 95% CI or number of participants analyzed

This outcome will be selected upon completion of data extraction for study 1, which will allow us to map the outcomes frequently investigated in the sample. This outcome must meet the aforementioned requirements as these will be necessary to conduct GRADE assessments necessary for study 2. Given that oral health SRs have been found to be most frequently downgraded in the study limitations and imprecision domains, [9] the aforementioned criteria are the minimum that our review team will require to conduct GRADE assessments. We selected a single outcome that is most frequently investigated to make it feasible for our review team to conduct GRADE assessments.

#### Eligibility criteria

##### Inclusion criteria

SRs eligible for this study must meet all of the eligibility criteria outlined above for study 1, in addition to reporting on the outcome of interest.

#### Data extraction

All data will be extracted independently and in duplicate using a piloted data extraction form. Reviewers will begin extraction upon completion of a calibration exercise. For all eligible SRs, we will extract the SRs’ conclusion for the outcome of interest alongside additional data including whether the conclusions made by study authors relied on statistical significance, included recommendations, and considered if there were any limitations. For SRs not using GRADE, we will extract the minimum information needed for our team to make a GRADE assessment. For SRs where a meta-analysis was conducted for the outcome of interest, this will include the results of the meta-analysis. In cases where the outcome of interest is summarized without a meta-analysis, we will extract the list of RCTs analyzed for the outcome, the number of participants analyzed overall, the SRs’ narrative summary of the analysis, and any effect estimates provided for each of the individual RCTs. In the case where the outcome of interest was measured at multiple time points or investigated for multiple comparisons, we will only consider the results of the shortest time point and the first comparison listed in the results. We will also extract the results of the RoB assessment, identify the level of contextualization used to assess imprecision, and determine whether there was any evidence of publication bias or indirectness for the outcome of interest. The additional data extraction fields for this study can be found in Table 4.

**Table 4:** Additional data extraction fields for the outcome of interest in study 2

Section	Data to be extracted
Results	• Did the SR authors conduct GRADE assessments for the outcome of interest? (yes/no) If the review authors did not use GRADE, extract the following: • How do the authors define the outcome of interest and at what time point is it measured? • What intervention and comparator are being investigated for the outcome of interest? • What tools are used to measure the outcome of interest (i.e., specific scale, number of teeth)? • If a meta-analysis was completed extract: ◦ The type of effect measure used by the authors ◦ The pooled effect estimate and 95% CI ◦ Screenshot of the forest plot ◦ If there is no forest plot, also extract the *I*^2^ value and corresponding *p*-value • If there was no meta-analysis extract: ◦ The included RCTs used to analyze the outcome of interest (extract first author name and reference number) ◦ The number of participants analyzed for the outcome of interest ◦ Verbatim quotation of the qualitative synthesis of the outcome of interest by the SR authors ◦ Any effect estimates provided for each of the included RCTs investigating the outcome of interest • Was a minimally or partially contextualized approach used to assess imprecision? • Results of the RoB assessment • Is there evidence of serious or very serious indirectness? (yes/no) ◦ If yes, provide a rationale. • Is there any reason to suspect publication bias? (yes/no) ◦ If yes, provide a rationale.
Conclusions	• Verbatim quotation of the SR conclusion statements pertaining to the outcome of interest from the SRs conclusion section ◦ If there is no conclusion section, this is to be extracted from the discussion section. If there is no conclusion outlined in the discussion, this is to be extracted from the abstract. • Do the authors rely on a *p*-value to make their conclusions (e.g., *p* < 0.05)? (yes/no) • Do the authors make any recommendations in their conclusion for the outcome of interest? (yes/no) • Do the authors consider if there are any limitations to their findings in their conclusion (by means of a GRADE assessment or otherwise)? (yes/no)

#### Data analysis

We will use a study selection flow chart to present the retrieved articles and tables to summarize the characteristics of eligible studies.

*To determine whether a lack of certainty of the evidence assessments is a predictor of inappropriately formulated conclusions in SRs (objective A)*, we will first evaluate the conclusions made by all the SRs for the outcome of interest, irrespective of whether they use GRADE. Two reviewers will independently assess these conclusions to determine whether they are appropriately formulated, and conflicts will be resolved through discussion or by a third reviewer where needed. Once all conclusions have been classified as appropriately formulated or not, we will use the odds ratio and its 95% CI to evaluate whether SRs using GRADE are more likely to formulate appropriate conclusions compared to SRs not using GRADE.

A conclusion will be considered to be appropriately formulated if it meets all the following criteria:

- The conclusion is justified by the results presented in the review.
- The conclusion does not rely on statistical significance [21].
- The conclusion considers if there are any limitations.
  - We will consider SR authors to have addressed limitations by stating whether or not the results are impacted by any number of factors (e.g., low quality of RCTs, heterogeneity, small sample size, publication bias, short follow-up time in RCTs) or referencing their GRADE certainty of the evidence rating.
- The conclusion does not make recommendations [22].

*To determine whether the use of GRADE changes the conclusions of dentistry SRs (objective B)*, reviewers will evaluate the conclusions of a subset of the study sample which does not utilize the GRADE approach and report on the outcome of interest. First, we will classify the authors’ conclusions in terms of their certainty as either definitive or recognizing uncertainty (addresses any limitations of the evidence by means of a GRADE assessment or through some other means). Examples of other ways to recognize uncertainty include stating that the results should be interpreted with caution due to high risk of bias, heterogeneity, small sample size, etc., stating that there were limitations in the evidence, stating that there is insufficient evidence to draw a conclusion, or stating that further high-quality studies are needed. We will also identify how the effect size is categorized in the SR conclusions according to the level of contextualization used by the authors of each SR (i.e., minimally contextualized or partially contextualized) [23]. A minimally contextualized approach will be defined as an approach that focuses on whether an important effect exists (i.e., negligible/trivial/no difference or important difference between interventions), while a partially contextualized approach defines the magnitude of the effect (i.e., negligible, small, moderate, or large) [23]. We will assume a minimally contextualized approach is used unless the authors explicitly state using a partially contextualized approach or if this can be inferred from their conclusions as they refer to different magnitudes of effect. If the SR authors rely on statistical significance to classify the effect size, we will consider this to be a minimally contextualized approach. Conclusions will be classified by two reviewers until a consensus is reached.

Second, after classifying the authors’ original conclusions, the review team will complete the GRADE assessments for the outcome of interest in these SRs independently and in duplicate. We will assess the imprecision using the same level of contextualization used by the authors of each SR (i.e., minimally contextualized or partially contextualized) as classified by our team using the criteria above. We will use the information provided by the SR authors to assess the risk of bias and inconsistency. We will assume no concerns for indirectness and publication bias unless otherwise stated in the SRs’ results or discussion sections. Using the results of our GRADE assessment, we will then formulate one conclusion per SR as shown in Table 5, using the same level of contextualization utilized by the SR authors (Table 5) [24].

**Table 5:** Methods for formulating conclusions

Level of certainty	Conclusion based on a minimally contextualized approach	Conclusion based on a partially contextualized approach
**High**	Intervention X increases/decreases the outcome by an important/negligible amount when compared to intervention Y.	Intervention X increases/decreases the outcome by a negligible/small/moderate/large amount when compared to intervention Y.
**Moderate**	Intervention X probably increases/decreases the outcome by an important/negligible amount when compared to intervention Y.	Intervention X probably increases/decreases the outcome by a negligible/small/moderate/large amount when compared to intervention Y.
**Low**	Intervention X may increase/decrease the outcome by an important/negligible amount when compared to intervention Y.	Intervention X may increase/decrease the outcome by a negligible/small/moderate/large amount when compared to intervention Y.
**Very low**	Intervention X may increase/decrease the outcome by an important/negligible amount when compared to intervention Y, but the evidence is very uncertain.	Intervention X may increase/decrease the outcome by a negligible/small/moderate/large amount when compared to intervention Y, but the evidence is very uncertain.

After this, our team’s conclusion will be compared to the authors’ conclusions with respect to the certainty and effect size (Table 6). If the review authors’ original conclusion only states that there is insufficient evidence to comment on the outcome or does not comment on the effect size, we will only evaluate whether the conclusion changes with respect to certainty. We will calculate the percentage of conclusions which changed after a GRADE assessment was completed with respect to the classification of the certainty and/or effect size. We will also calculate the percentage of conclusions which have increased certainty, decreased certainty, increased effect size, or decreased effect size after a GRADE assessment is conducted. This will allow us to evaluate how the utilization of GRADE may impact authors’ conclusions by assessing whether conclusions change following GRADE assessments and by determining the direction of that change (i.e., do conclusions become more or less conservative following GRADE assessments?).

**Table 6:** Methods for determining the effect of the GRADE approach on SR conclusions

		Classification of SR authors’ conclusion	Classification of our conclusion (after GRADE assessments)	Effect of our use of the GRADE approach on the SR conclusion
**Certainty**		Definitive	High	No change
			Moderate, low, or very low	The level of certainty of the SR conclusion decreased
		Recognizing uncertainty	High	The level of certainty of the SR conclusion increased
			Moderate, low, or very low	No change
		Recognizing uncertainty^a^	High, moderate, or low	The level of certainty of the SR conclusion increased
			Very low	No change
**Effect size**	Minimally contextualized approach	Negligible effect	Negligible effect	No change
			Important effect	The magnitude of the effect in the conclusion of the SR increased
		Important effect	Negligible effect	The magnitude of the effect in the conclusion of the SR decreased
			Important effect	No change
	Partially contextualized approach	Negligible effect	Negligible effect	No change
			Small, moderate, or large effect	The magnitude of the effect in the conclusion of the SR increased
		Small effect	Negligible effect	The magnitude of the effect in the conclusion of the SR decreased
			Small effect	No change
			Moderate or large effect	The magnitude of the effect in the conclusion of the SR increased
		Moderate effect	Negligible or small effect	The magnitude of the effect in the conclusion of the SR decreased
			Moderate effect	No change
			Large effect	The magnitude of the effect in the conclusion of the SR increased
		Large effect	Negligible, small, or moderate effect	The magnitude of the effect in the conclusion of the SR decreased
			Large effect	No change

^a^This refers to the conclusions where it is only stated that there is a lack of sufficient evidence

## Ethics and dissemination

Ethics committee approval and consent are not required for any component of this methodology project since only previously published data will be used.

## Discussion

This methodological study will reveal the frequency of GRADE assessments in dentistry SRs and investigate whether the use of GRADE has an important impact on the conclusions of a SR. The findings of this study may be compared to similar studies conducted in the past in order to determine whether the uptake of GRADE has increased in the past 5 years [9, 10]. Thus, this study will increase awareness regarding the quality of the current dental SRs, particularly with regard to how they formulate the conclusions. This can help inform SR consumers of the potential limitations of SRs and how to appropriately interpret and evaluate a SR’s conclusions.

By evaluating the quality and frequency of GRADE assessments in dentistry SRs, and whether appropriate conclusions are being formulated, this study will also highlight any shortcomings observed in the current dental SR literature. This may help improve dentistry SRs by informing SR authors of common flaws and how to address them. One potential implication for this research is to elucidate the main limitations in dentistry SRs, which could allow us to develop a guidance article specifically for dentists on the application of GRADE. This study will also describe the certainty of evidence reported by SRs in the dental field, which will allow us to identify where more high-quality randomized trials may be needed to strengthen the body of evidence.

Based on a previous study evaluating a sample of 91 oral health SRs of which most did not use the GRADE approach, we anticipate that this study will have similar findings [9]. Additionally, this previous study found that the SRs that did use GRADE found the certainty of the evidence to be mostly low or very low [9]. We believe that our study will have similar findings which can help inform dentistry SR authors and consumers on the methodological strengths and limitations of the current literature as well on the impact of GRADE use on formulating appropriate conclusions. For instance, if we find that few SRs use GRADE, but that these SRs are more likely to be of higher methodological quality and formulate appropriate conclusions, this may prompt dentistry SR authors to use the GRADE approach and to improve the overall quality of their SRs. If, however, we find a high frequency of GRADE use, we can better evaluate whether GRADE is being used appropriately, which could provide SR authors guidance on future GRADE use. In either case, SR consumers would gain insight into whether current SRs and their conclusions are trustworthy.

### Strengths and limitations

The strengths of this study include its high methodological quality, including a search strategy that maximizes specificity, specific eligibility criteria, and duplicate screening and data extraction. Another strength of this methodological study is the use of piloted data extraction forms accompanied with detailed instructions for reviewers as well as reproducible appraisals of methodological rigor due to the use of the well-established ROBIS tool. However, in order to ensure reproducible assessments, we evaluated methodological quality using two of the most objective aspects of the ROBIS tool as they were not dependent on each SRs’ clinical question. While we considered focusing on SRs investigating a specific clinical question, we chose to prioritize a comprehensive overview of various topics and thus chose ROBIS domains most applicable to our objectives.

Another limitation of this study is that we will not search for SRs in grey literature sources. Since grey literature is not peer-reviewed and has been found to be of lower methodological quality than peer-reviewed literature, this may bias the results of this study to be more positive [25]. Additionally, we will only retrieve studies from one electronic database, MEDLINE. However, we believe it is unlikely that the database in which journals are indexed is related to our question, and thus, we do not think SRs retrieved via MEDLINE will be qualitatively different from those in another database. Also, while our study will only include SRs from 2016 onwards that included RCTs, we do not think this is an important limitation as we intended to allow for sufficient time for the adoption of GRADE considering the first set of guidelines was published in 2011 and that additional guidance for SRs of observational studies has been provided as recent as 2019 [16–20, 26]. Additionally, as RCTs generally result in higher quality evidence, we considered them to be most relevant to dentists.

Additionally, the quality of our GRADE assessments will be contingent on the quality of the information reported by the SR authors. For example, our rating for the RoB domain will be solely based on the RoB assessments of the SR authors, regardless of whether they have used a validated tool. In addition, as we anticipate poor reporting quality, we have planned not to analyze title/abstract and full-text screening separately as per the ROBIS tool; instead, we will analyze the methods for screening overall. Nonetheless, the large number of studies to be included in this review will provide a relatively accurate perspective on the use of GRADE in recent dentistry SRs. Furthermore, we have established various protocols to limit the number of judgments made by reviewers. This includes specific eligibility criteria, piloted data extraction forms, and evidence-based criteria for assessing the appropriateness of GRADE assessments and SR conclusions. To minimize any further subjectivity in the process, we will complete all steps independently and in duplicate, and disagreements will be discussed with a third reviewer.

## Conclusion

This methodological study will provide insight into the extent to which GRADE assessments affect the conclusions of SRs in dentistry. It will also investigate the frequency of GRADE in dentistry SRs and whether SR authors use GRADE appropriately and use it to inform their conclusions. This study aims to inform SR readers and authors on the impact of GRADE assessments on the conclusions and methodological quality of a SR.

## Supplementary Information

**Additional file 1.** PRISMA-P Checklist. PRISMA-P checklist outlining which components were adhered to in the formation of this protocol and where in the manuscript they can be found.

## Data Availability

Not applicable.

## Abbreviations

SR: Systematic review
RoB: Risk of bias
ROBIS: Risk of Bias Assessment Tool for Systematic Reviews
AMSTAR: Assessing the Methodological Quality of Systematic Reviews
GRADE: Grading of Recommendations, Assessment, Development, and Evaluation
RCT: Randomized controlled trial
CI: Confidence interval
